# Translational Impact of Genetics and Epigenetics of CGRP System on Chronic Migraine Treatment with Onabotulinumtoxin A and Other Biotech Drugs

**DOI:** 10.3390/toxins17070355

**Published:** 2025-07-17

**Authors:** Damiana Scuteri, Paolo Martelletti

**Affiliations:** 1 Department of Health Sciences, University “Magna Graecia” of Catanzaro, 88100 Catanzaro, Italy; 2 School of Health, Unitelma Sapienza University of Rome, 00161 Rome, Italy

**Keywords:** migraine, onabotulinumtoxin A, epigenetics, serum miRNAs, DNA methylation, CGRP, anti-CGRP-R monoclonal antibodies, gepants

## Abstract

Migraine is a neurovascular paroxysmal disorder characterized by neurogenic inflammation and has a remarkable impact on the quality of life. The Food and Drug Administration (FDA) approved onabotulinumtoxin A in 2010 for the prophylactic treatment of chronic migraine. Today, in its 4th decade, it is approved in 100 countries for 15 main indications. Its mechanism of action, based on the inhibition of neurotransmitter release from primary sensory neurons, is very complex: it affords antinociception, but it also has an analgesic effect on neuropathic pain conditions and reduces the need for rescue medications. Genetic variants have been investigated for their potential role in the pathogenesis and clinical expression of migraine and of the response to treatments. These studies primarily involved genes associated with vascular regulation and cardiovascular pathology, including those encoding angiotensin-converting enzyme (ACE) and methylenetetrahydrofolate reductase (MTHFR). However, epigenetics and, particularly, genetic and epigenetic modifications are still poorly studied in terms of understanding the mechanisms implicated in susceptibility to migraine, aura, chronification and response to symptomatic and preventive treatments. In particular, the aim of the present study is to gather evidence on the genetic variants and epigenetic modifications affecting the pathway of the calcitonin gene-related peptide (CGRP), the target of onabotulinumtoxin A and of all the novel monoclonal antibodies.

## 1. Introduction

Migraine, recognized as a neurovascular disorder, represents a worldwide and prevalent leading cause of disability affecting 15.2% of the global population [1]. It is one of the leading causes of years lived with disability [2], and its impact is even more burdensome due to its prevalence in those of working age. Indeed, it is classified, based on the days per month in which the patients experience attacks, as episodic (fewer than 15 migraine or headache days) or chronic (at least 15 days, among which 8 or more are migraine days) [3]. The occurrence of attacks can persist over many years, as the profile changes over the life span [4]. Nevertheless, the frequency and intensity of attacks often decrease with age, despite specific cases often being neglected and undertreated [5,6]. In fact, migraine is a chronic disorder characterized by episodic manifestations due to recurrent attacks, presenting some crossover with epilepsy [7] but with a very complex physiopathology. A remarkable, interesting feature of this disease is the presence of aura, consisting of reversible neurological and mainly visual symptoms occurring for 5–60 min in 30% of cases [1,3,8] and usually preceding attacks lasting 4–72 h, which are unilateral, pulsating and of moderate to severe intensity. Bidirectional transitions between episodic and chronic migraine are observed [9], reflecting the dynamic and multifaceted nature of migraine phenotypes. However, long-duration primary chronic daily headaches represent one of the most important risk factors for progression and transformation into chronic migraine [9,10].

Accordingly, the comprehensive characterization of at-risk patients is of the utmost importance, demanding objectively measurable biomarkers. Within this complex and evolving frame, epigenetics, including gene-specific DNA methylation patterns and several novel mechanisms such as circulating microRNA (miRNA) expression, has been under investigation. In particular, studies on the latter have been gaining increasing interest over the years because genetics and risk factors do not fully explain susceptibility to the development of migraine, aura and chronification, as well as the response to pharmacological treatments [11]. Botulinum neurotoxin type A was demonstrated to be effective across a range of pain syndromes [12,13]. Its effectiveness in pain relief relies on the mechanism of inhibition of the exocytosis of neurotransmitters and neuropeptides involved in pain and processing [14], through the cleavage of the 25 kDa synaptosomal-associated protein (SNAP-25) [15]. In 2010, onabotulinumtoxin A received approval from the U.S. Food and Drug Administration (FDA) for the prevention of chronic migraine [16]. This approval was based on the results of the Phase III PREEMPT (Phase III Research Evaluating Migraine Prophylaxis Therapy) I and II clinical trials (NCT00156910, NCT00168428), which set the rationale for its use and the recommended mode and dosage of administration in chronic migraine patients [17,18,19]. Hence, the approval of onabotulinumtoxin A (BTX-A) began the Biotech Era of chronic migraine treatment, subsequently expanded with monoclonal antibodies (mAbs) targeted towards the signaling of calcitonin-gene related peptide (CGRP) [20]. The aim of the present review paper is to shed light on the genetics and epigenetics of the pathways in which the neuropeptide CGRP is involved in relation to the efficacy and safety of BTX-A.

## 2. Epigenetic Mechanisms and Migraine

Since migraine is a life-span-transforming neurological disorder, it is an evolutive chronic condition characterized by functional and anatomical changes during the process of chronification and displays an age-dependent change rate [21]. The new term “epigenetics” was proposed by Conrad Hal Waddington in 1942. Nowadays, the term epigenetics refers to heritable and stable changes in the expression of genes, occurring by means of alterations in the chromosome rather than in the DNA sequence [22]. Alterations to histone proteins (acetylation and transcriptional activation-related methylations) around which the DNA is packaged facilitate gene expression, while methylation in the CpG islands of promoter sequences induces gene silencing. However, the transcriptional repression of histone methylation has also been observed, and other mechanisms are represented by histone ubiquitination, phosphorylation and lysine β-hydroxybutyrylation along with noncoding RNAs and nucleosome remodeling. Noncoding RNAs (ncRNAs), in which miRNAs are included together with small interfering RNAs (siRNAs) and long ncRNAs, are RNA molecules that do not undergo translation into proteins. These features can allow them to play the potentially pivotal role of future biomarkers to help in diagnosis and as predictors of response to the treatment of the disorder. Also, circular RNAs (circRNAs), a type of ncRNAs without a 5′ cap or 3′ poly (A) tail, are thought to be involved in synaptic function [23]. Furthermore, Ten-eleven translocation (TET) family proteins (TETs) can oxidize 5-methylcytosine (5mC) iteratively, and the dysregulation of their action may be involved in several diseases [24].

A recent study investigated the expression of a wide panel of serum miRNAs in patients affected by migraine and healthy controls. The aim of the study consisted of searching for differences between patients affected by migraine and healthy controls within ictal and interictal phases but was also related to the occurrence of aura and to the frequency of migraine attacks [25]. The latest genome-wide association studies (GWASs) underlined the association between 44 single-nucleotide polymorphisms of genes involved in molecular pathways related to vascular function or metal ion homeostasis with migraine without aura (see [21]). One GWAS concerned with chronification demonstrated the occurrence of two CpG sites in the SH2D5 and NPTX2 genes implicated in the regulation of synaptic plasticity (see [21]).

## 3. Influence of Environment on Migraine Susceptibility and Features

The epigenetic modulation of migraine headaches may represent a fundamental tool to add to the pharmacotherapeutic machinery in clinics since epigenetic modifications are usually reversible. This mechanism can be one of the most important among the several mechanisms at the root of use in the clinical management and prevention of chronic migraine. Epilepsy and migraine share pathophysiological features at the root of clinical characteristics, making patients affected by one more predisposed to the other; miR-22, miR-34a, miR-155, miR-211 and Let-7b were suggested to be associated with both diseases [26]. miR-26a-5p and miR-145-5p were upregulated in the serum of patients affected by migraine. Also, miR-19a-3p was downregulated during headache compared to the interictal period. However, further validation in a larger population is needed. The cross-sectional controlled study NCT05891808 focusing on monocytes proved that the expression of miR-155 is higher in patients suffering from chronic migraine with medication overuse headache (MOH) than in patients with episodic migraine and in episodic patients affected by migraine rather than in healthy controls [27]. Also, a correlation with increased levels of interleukin (IL)-1β and tumor necrosis factor (TNF)-α proteins was found. The present results agree with the widely reported dysregulation of ILs in patients affected by migraine [28]: confirmatory studies with a larger sample size for the exploratory outcomes assessed are needed.

The role of intrinsic and environmental factors in the epigenetics of migraine and response to preventative treatments is a new aspect under investigation. An exploratory study compared miRNA levels in the peripheral blood mononuclear cells of patients with migraine to healthy controls in a cohort of non-menopausal women [29]: the data showed the differential expression of miR-342-3p, miR-532-3p and miR-758-5p. The results deserve further investigation in a larger and more heterogeneous sample. Valproate is a histone deacetylase (HDAC) inhibitor involved in the facilitation of chromatin remodeling, and its effectiveness in migraine may be, at least in part, due to its effects on the epigenome. This clinical evidence is strengthened by studies conducted in animal models for depression, proving that antidepressant treatment requires reduced HDAC activity to exert effects on chronic stress [30]. The data gathered support the use of epigenome modulation, which represents a novel remarkable target in migraine treatment. In fact, the clinical relevance of epigenetic studies in the field of migraine will encompass diagnosis, explaining, partly, the susceptibility and pathophysiology of migraine but also therapy and the fight against resistance to the available pharmacological armamentarium. One of the most recent examples of the importance of epigenetics in the clinical setting of migraine is represented by the longitudinal epigenome-wide association study within the “Chronification and Reversibility of Migraine clinical trial”. It highlights a longitudinal reduction in HDAC4 DNA methylation associated with treatment response and points at baseline MARK3 DNA methylation as an early biomarker for responders, suggesting that these modifications represent novel pharmacotherapeutic targets [31].

## 4. CGRP Pathway Modifications

**Figure 1:**
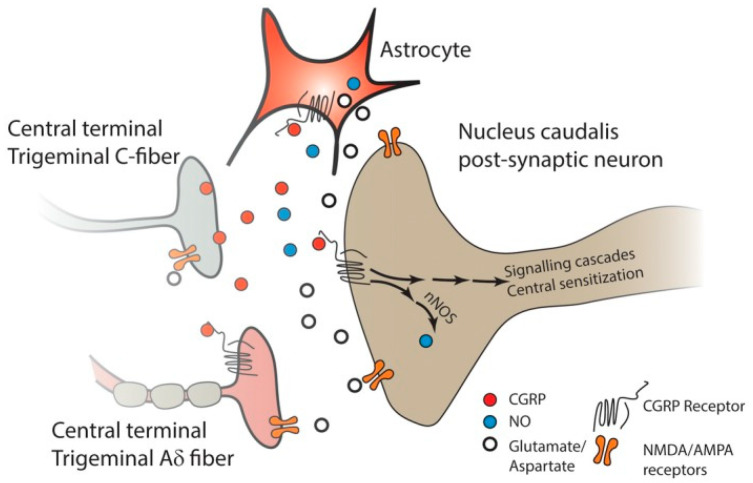
The CGRP released from the central terminals of unmyelinated nociceptive C-fiber TG neurons can activate the CGRP receptors of the second-order neurons and elicit the production of NO via nNOS. NO acts as a retrograde neuromodulator and enhances the activity of both the CGRP and non-CGRP nerve terminals synapsing with the second-order neuron, resulting in enhanced transmitter release. The CGRP released from the TG neuron can also activate astrocytes, eliciting the release of NO and other inflammatory mediators, and act on receptors on the terminals of neighboring Aδ neurons that express the CGRP receptor, leading to their sensitization. Moreover, the release of excitatory transmitters such as glutamate from second-order neurons and astrocytes results in the activation of NMDA and AMPA receptors on second-order neurons, on primary afferent nerve terminals and on astrocytes to further promote the release of excitatory substances, thus further enhancing the activity of the second-order neuron and leading to a state of central sensitization. α-amino-3-hydroxy-5-methyl-4-isoxazolepropionic acid receptor, AMPA-R; calcitonin gene-related peptide, CGRP; N-methyl-D-aspartate, NMDA; nitric oxide, NO; neuronal nitric oxide synthase, nNOS; trigeminal ganglion, TG (reproduced with permission from [33]).

Another fundamental issue remarkably increasing the social burden of migraine is represented by refractoriness even to the most novel treatment options targeting the calcitonin gene-related peptide (CGRP) pathway, i.e., BTX-A; receptor antagonists, named gepants; and monoclonal antibodies (mAbs). In fact, predictors of the efficacy of BTX-A in chronic migraine patients were investigated through an assessment of allelic and genotypic frequencies and the dominance/recessivity hypothesis of the allelic variants [32]. The role of CGRP in migraine pathogenesis is illustrated in Figure 1 (reproduced with permission from [33]).

Two polymorphisms associated with differences in response to BTX-A were identified: CALC A, the gene encoding CGRP, rs3781719, with allele C present at 26.9% in responders and at 40.9% in non-responders (*p* = 0.007; OR = 3.11 (1.33–7.26)), and TRPV1 rs222749, with allele A representing 4.17% in responders and 12.5% in non-responders (*p* = 0.013; OR = 3.29 (1.28–8.43)) [32] (Table 1).

These data are in agreement with the epidemiology of non-response even to the most novel anti-CGRP/R mAbs. In fact, the initial response to anti-CGRP mAbs is not consistent across time, and its worsening might be linked to anxiety and depression with noteworthy clinical repercussions. Moreover, up to 40% of patients in clinical practice experience the failure of treatment with no evident predictor of 100% response in patients suffering from high-frequency episodic migraine or chronic migraine [34]. According to the need and ethical principle to reduce the risks associated with treatment, the switch to gepants is a very interesting option. Their effectiveness can be explained by the action possibility of rimegepant and atogepant as antagonists of the amylin AMY1 receptor with high affinity for amylin, which when activated can be responsible for resistance to mAbs [35]. The study NCT04659226 evaluated the expression levels of circulating miRNAs with a potential role as epigenetic biomarkers of treatment response before and after treatment with erenumab [36]. The 16 bp deletion in the first intron of the CALC A gene, with triplet G-run motifs, was investigated for its association with the risk of migraine in an Australian population, without providing any significant results [37]. Moreover, the rs145837941 genotype in the coding sequence of the CALC A gene might alter the structure of the propeptide [38]. The SNPs rs3754701 and rs7590387 of the gene encoding RAMP1 were associated with migraine in a genome-wide association study [39], but the results are unpublished. Most studies investigating the genetics and epigenetics of the CGRP pathway failed to find significant associations. An exploratory case–control study found that patients with episodic migraine present a lower methylation level in two CpG sites at the proximal promoter region of the CALC A gene, and a significant correlation of the DNA methylation level at different CpG sites with several clinical characteristics of the disease was reported [40]. The gathered results highlighted the role of hsa-miR-143-3p levels in the response to the block of the CGRP receptor induced by erenumab. Furthermore, the levels of hsa-miR-34a-5p and hsa-miR-382-5p vary according to the type of migraine and during the follow-up. A systematic review performed following the guidance from the Human Genome Epidemiology Network for reporting gene–disease associations identified two recurring polymorphisms affecting the CGRP pathway. These are the rs3781719 (T > C) SNP of CALC A and the rs7590387 SNP of the gene encoding the receptor activity-modifying protein (RAMP) 1 (C > G) (see [41]). Nevertheless, the heterogeneity between and across studies and the small sample size do not allow us to draw definite conclusions based on the Human Genome Epidemiology (HuGE) systematic reviews and meta-analyses’ risk-of-bias score for genetic association studies [41]. In fact, the genotype characterized by the rs7590387 G allele of the RAMP1 gene was associated with a lower probability of being a 75% responder to erenumab, i.e., a patient that shows a reduction in monthly migraine days ≥75%. Furthermore, the modifications affecting the complex heterotrimeric G-protein-coupled receptor activated by CGRP, made up of the calcitonin-like receptor (CLR), RAMP1 and the small receptor component protein (RCP), have been poorly studied in genetic and epigenetic association studies so far. Different expression levels and organizations of the receptor might be involved in non-response.

## 5. Discussion

**Figure 2:**
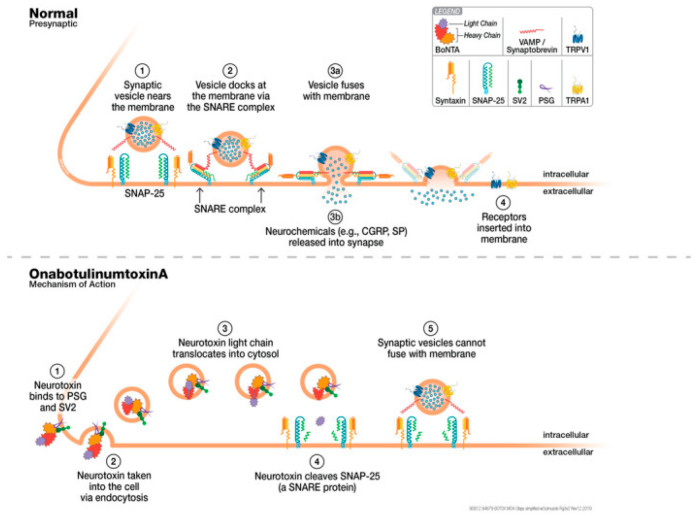
The mechanism of onabotulinumtoxin A (BTX-A) at the synapse. The top panel shows the fusion of large dense core synaptic vesicles with the nerve terminal membrane in the absence of BTX-A. By step 4, the neurotransmitters contained in the synaptic vesicles are released into the synapse, and receptors/ion channels are inserted into the nerve terminal membrane. The bottom panel shows the steps of BTX-A action at nerve terminals. The end result is that synaptic vesicles cannot fuse with the nerve terminal membrane, preventing the release of neurotransmitters at the synapse and inhibiting the insertion of receptors/ion channels into the nerve terminal membrane (reproduced and adapted with permission from [43]).

Migraine is a chronic paroxysmal neurological disorder characterized by primary moderate to severe headache, frequently accompanied by a constellation of reversible neurological and systemic symptoms, including photophobia, phonophobia, cutaneous allodynia, nausea, vertigo and dizziness [8]. The role of genetic and epigenetic modifications in the susceptibility to migraine, the high-frequency of attacks and chronification, aura and the response to treatments such as BTX-A still deserves deepened investigation. In fact, BTX-A in migraine is thought to depend on the proteolysis of the protein SNAP-25, required for the membrane fusion that is needed for the exocytosis of CGRP and various other neuropeptides and neurotransmitters [42] (Figure 2, reproduced with permission from [43]).

This is a matter of the utmost importance particularly in situational prevention, consisting of the treatment of patients within the interictal phase when the risk for migraine attacks is increased [44], and it has a noteworthy impact on disability [45]. Polymorphisms and gene copy number variations (CNVs) of the functional receptor effectors Fc receptors (FcRs) were shown to be related to the efficacy of monoclonal antibodies, such as trastuzumab [46]. A HuGe systematic analysis indicated the rs7590387 SNP of RAMP 1 locus affecting the CGRP pathway as important for its role in migraine [41]. Furthermore, it can be involved in the transformation of episodic migraine into MOH [41]. Recently, the exploration of how dietary factors may impact migraine pathogenesis through epigenetic modifications has been hypothesized. Since folate is involved in DNA methylation, a role for a folate-rich “epigenetic diet” in the management of migraine was hypothesized; however, no results are conclusive enough to make a recommendation. Incidentally, CGRP may be involved in neural changes under the modulation operated by HDAC6, which may be related to migraine. The inhibition of HDAC6 causes a reduction in cortical-spreading depression and a block of the CGRP receptor [47]. However, this research line investigating the role of dietary factors suggests that diet could influence the gut–brain axis and could alter the expression of genes related to migraine susceptibility. Also, the role of genetic and epigenetic variations in the activity of Transient receptor potential cation channel subfamily A member 1 (TRPA1) deserves investigation in the response to botulinum neurotoxin type A and CGRP-targeted treatments. In fact, these channels are activated by a variety of migraine triggers, and the expression of the TRPA1 gene undergoes variations by DNA methylation, histone modifications, miRNAs, long ncRNAs and circular RNAs [48]. An retrospective Italian chart analysis carried out on treatments from February 2002 to January 2003 highlighted that 85% of patients experienced at least some degree of pain relief and reduced their use of analgesics with botulinum toxin [49], pointing at its importance. The mAbs approved between 2018 and 2020 by the FDA and the European Medicines Agency (EMA) for the preventive treatment of episodic and chronic migraine fostered a reduction in the use of BTX-A the field of migraine in spite of still present difficult-to-treat patients. A recent systematic review and meta-analysis appraising the safety of BTX-A in randomized, clinical studies demonstrated a higher occurrence of treatment-related adverse events (TRAEs) with oral topiramate, one of the most used preventative drugs. Moreover, the latter study highlighted the heterogeneity of the studies present in the literature (I^2^ = 96%; *p* < 0.00001) [50]. The data gathered in the present review point at the need for further adequately powered clinical trials and real-world studies to investigate the role of genetic and epigenetic modifications occurring along the CGRP pathway and the SNAP-25 complex to unravel the potential mechanisms of non-response. Furthermore, recent retrospective results highlight the neglected use of BTX-A [6,51,52]. Also, the role of BTX-A in the improvement of response to mAbs directed towards the signaling of CGRP has been highlighted. In fact, patients who switch to anti-CGRP mAbs after treatment with BTX-A are associated with a better clinical outcome [53].

Therefore, novel homogeneously designed studies of genetic association with larger and inclusive samples are mandatory to confirm and extend the results to the general population. In fact, existing gaps in research are related to heterogeneity among studies, small sample size and a lack of an adequate number of references for power calculation, not allowing us to detect true associations for small effects. Additional data from future studies may help in the prediction of responders based on treatment and be useful to establish sound epigenetic biomarkers for clinical decision. Hence, the role of gene expression and epigenetics deserves an in-depth study for the identification of reliable biomarkers for the diagnosis and prediction of response to treatment and to prompt trials investigating the efficacy and safety of novel pharmacological treatments. Next-generation artificial intelligence methodologies, proposed to facilitate the automation of routine clinical tasks, understand disease mechanisms and predict patients’ responsiveness to treatments, may be useful for clustering inhomogeneous data to dive into the still unrevealed mechanisms correlating with epigenetic modifications and the predisposition to the clinical and pharmacological features of migraine. Furthermore, studies assessing the effects of genetic and epigenetic modifications of a mAb, with a unique profile of fast action such as eptinezumab [54], also in combination with BTX-A [5,55], are needed to characterize the response of patients to these novel approaches.

## Figures and Tables

**Table 1:** Polymorphisms associated with differences in response to onabotulinumtoxin A (BTX-A).

Gene	Polymorphism	Effect	Reference
CALC A gene	rs3781719	allele C present at 26.9% in responders and at 40.9% in non-responders (*p* = 0.007; OR = 3.11 (1.33–7.26))	[32]
TRPV1 gene	rs222749	allele A representing 4.17% in responders and 12.5% in non-responders (*p* = 0.013; OR = 3.29 (1.28–8.43))	[32]

## Data Availability

No new data were created or analyzed in this study.
